# Supplementary figures and images for: Cardiology involvement and mortality in adult patients with advanced solid cancer complicated by atrial fibrillation
Source: PLoS One. 2025 Feb 25;20(2):e0319342. doi: 10.1371/journal.pone.0319342 (PMC11856317; doi:10.1371/journal.pone.0319342)

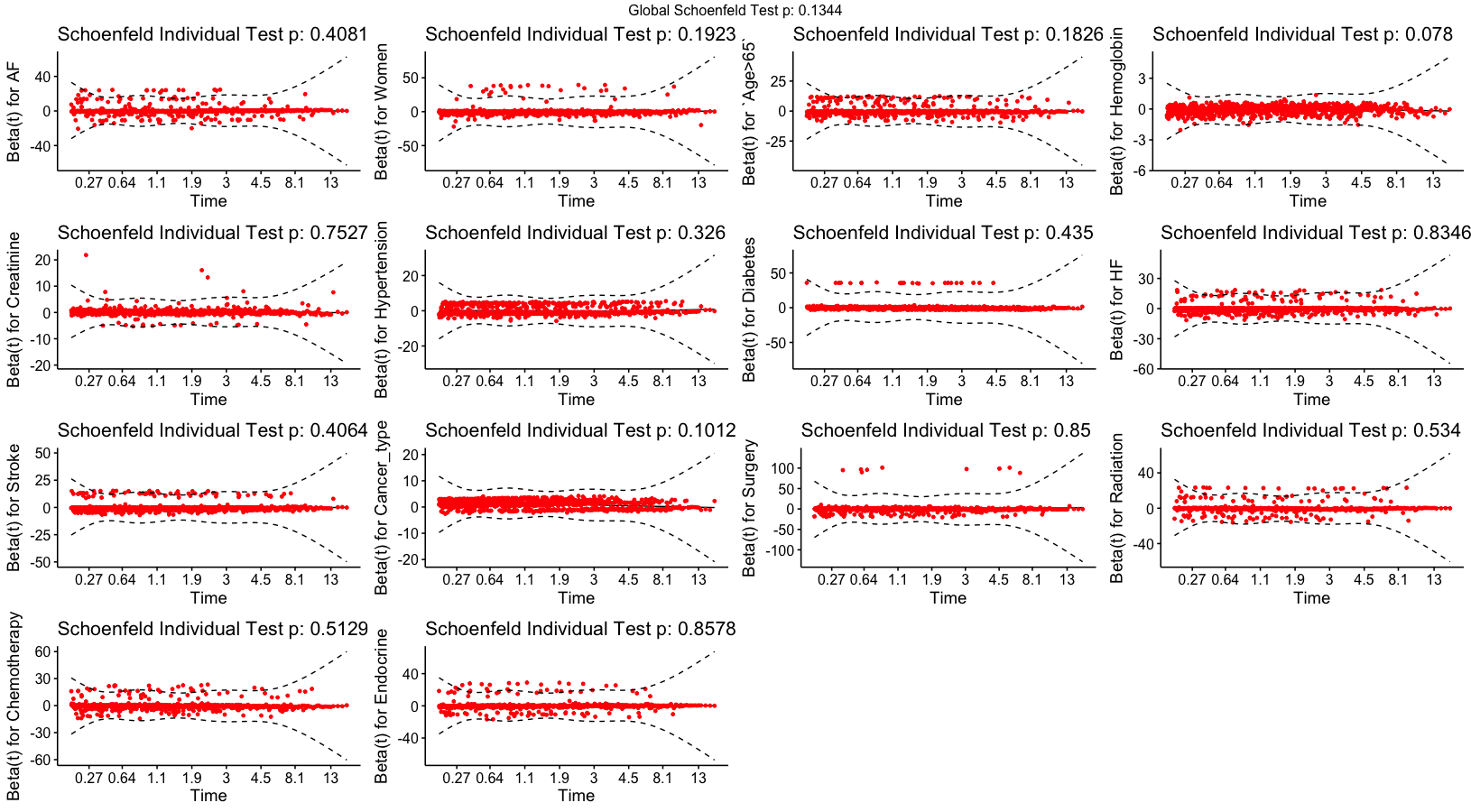

Supplement: S1 Fig — P values for all variables including the presence of AF, as well as that of the global Schoenfeld test were > 0.05, indicating that the proportional hazard assumptions were met. HF, heart failure. (Tiff) [file pone.0319342.s001.tiff]

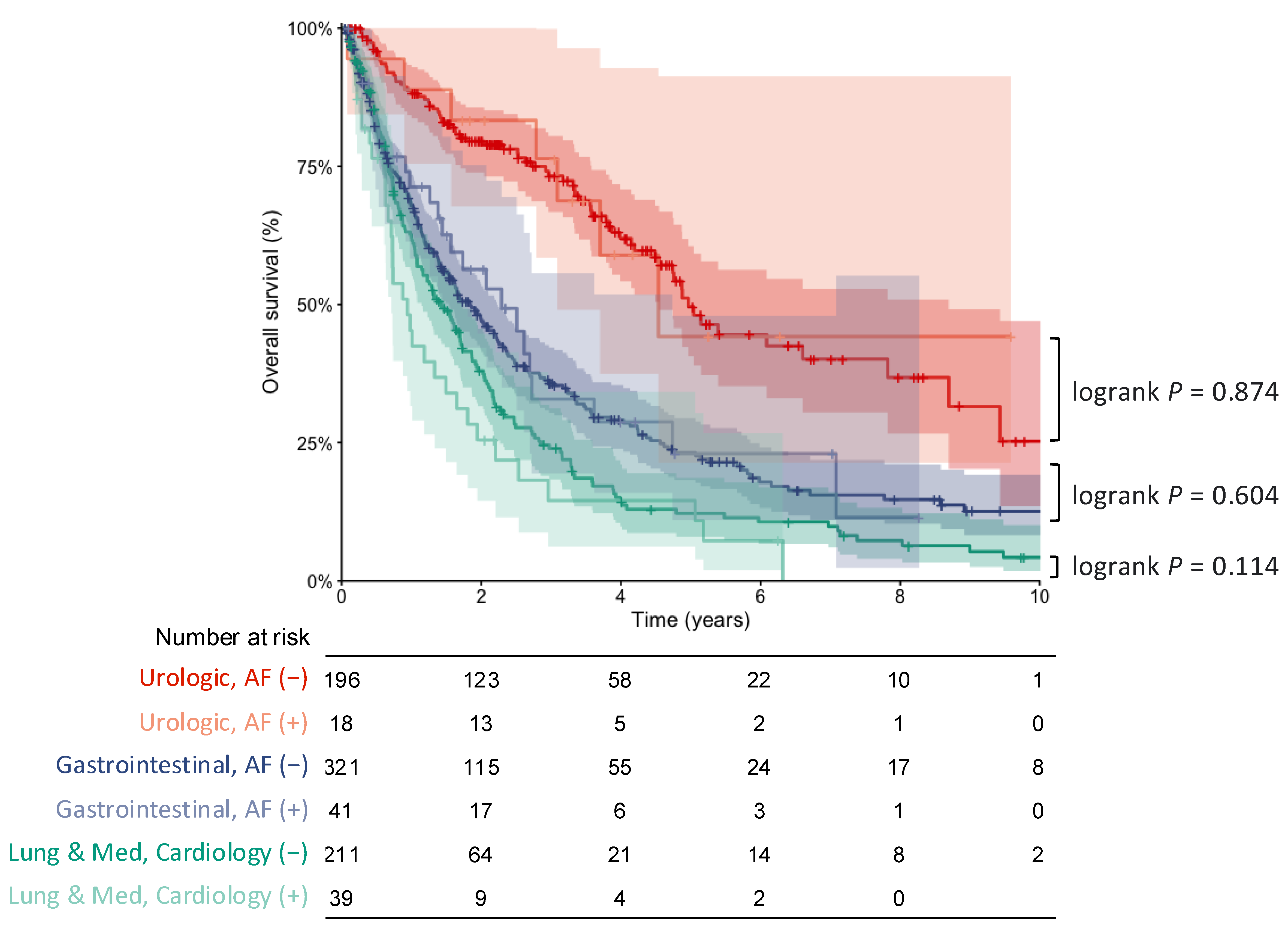

Supplement: S2 Fig — Patients with advanced urologic, gastrointestinal, and lung and mediastinal (lung and med) cancers were analyzed. Shaded zones indicate 95% confidence intervals. AF, atrial fibrillation. (Tiff) [file pone.0319342.s002.tiff]

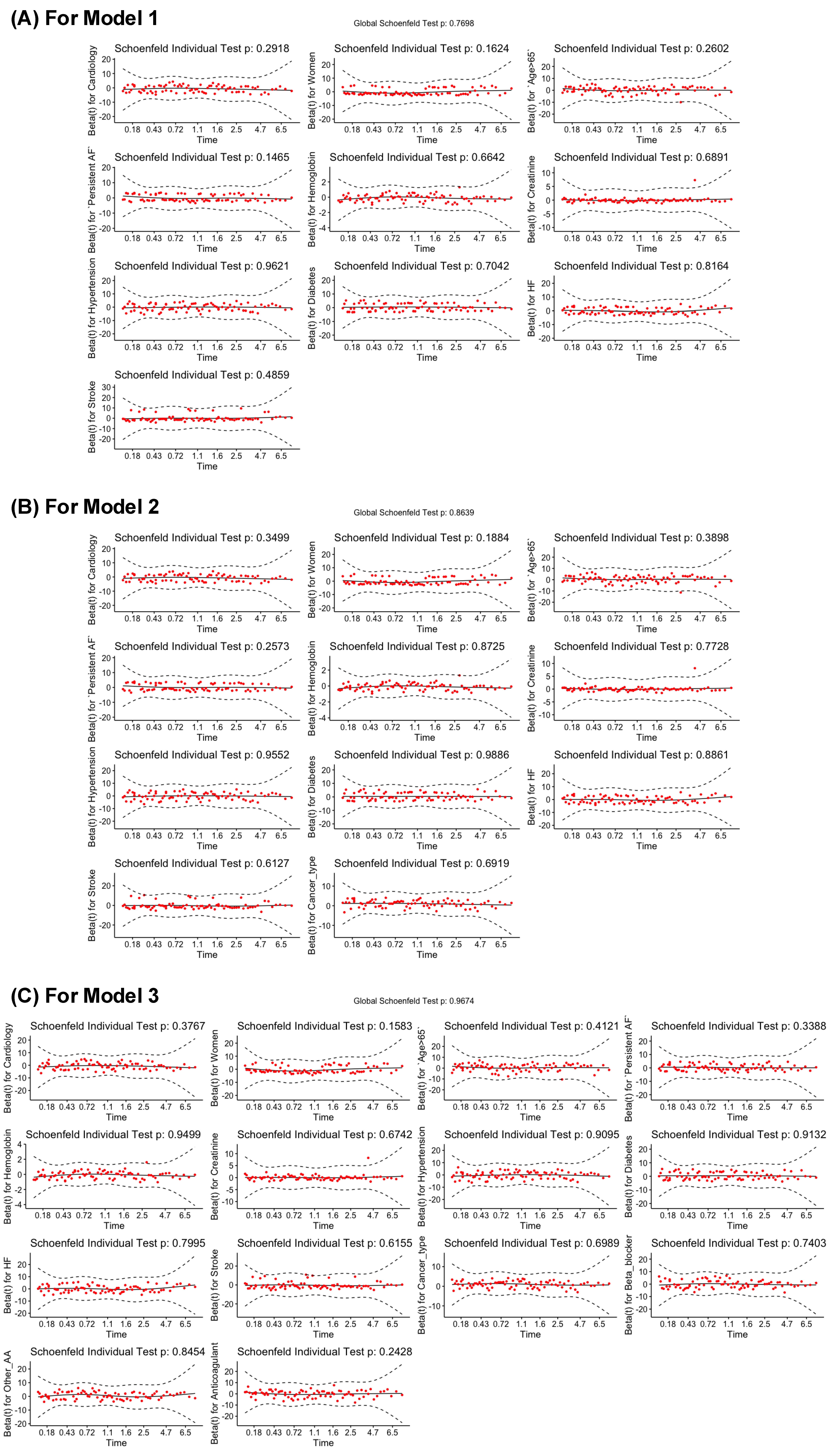

Supplement: S3 Fig — Panels A, B and C correspond to the scaled Schoenfeld residual plots for variables included in Models 1, 2 and 3 in Table 4, respectively. P value for each variable, as well as that for the global Schoenfeld test were > 0.05 in all models, indicating that the proportional hazard assumptions were met. AA, antiarrhythmics; AF, atrial fibrillation; HF, heart failure. (Tiff) [file pone.0319342.s003.tiff]
