# Supplementary material for: Cardiology involvement and mortality in adult patients with advanced solid cancer complicated by atrial fibrillation
Source: PLoS One. 2025 Feb 25;20(2):e0319342. doi: 10.1371/journal.pone.0319342 (PMC11856317; doi:10.1371/journal.pone.0319342)
Supplement: S1 Table — (DOCX) [file pone.0319342.s004.docx]

**S1 Table. Different treatment strategies among different cancer types in the entire cohort**

| **Total N = 1349** | **Urologic** | **Breast** | **Gastrointestinal** | **Lung and Mediastinal** | **Hepato-biliary-pancreatic** | **Other cancers ^*^** | **P value** |
| --- | --- | --- | --- | --- | --- | --- | --- |
|  | **(N = 214)** | **(N = 266)** | **(N = 362)** | **(N = 250)** | **(N = 162)** | **(N = 95)** |  |
| **Surgery** | 82 (38.3) | 32 (12.0) | 223 (61.6) | 15 (6.0) | 50 (30.9) | 71 (74.7) | < 0.001 |
| **Radiotherapy** | 34 (15.9) | 27 (10.2) | 30 (8.3) | 49 (19.6) | 8 (4.9) | 7 (7.4) | < 0.001 |
| **Chemotherapy** | 52 (24.3) | 199 (74.8) | 281 (77.6) | 232 (92.8) | 137 (84.6) | 55 (57.9) | < 0.001 |
| **Endocrine therapy** | 123 (57.5) | 126 (47.4) | 2 (0.6) | 0 (0) | 0 (0) | 23 (24.2) | < 0.001 |

Data are presented as number (percentage) and compared using χ^2^ test.

* Other cancers included gynecological cancer, thyroid cancer, oral cancer, head and neck cancer, sarcoma, and carcinoma of unknown primary.
