# Supplementary material for: Cardiology involvement and mortality in adult patients with advanced solid cancer complicated by atrial fibrillation
Source: PLoS One. 2025 Feb 25;20(2):e0319342. doi: 10.1371/journal.pone.0319342 (PMC11856317; doi:10.1371/journal.pone.0319342)
Supplement: S2 Table — (DOCX) [file pone.0319342.s005.docx]

**S2 Table. Different treatment strategies among different cancer types in patient with atrial fibrillation**

| **Total N = 122** | **Urologic** | **Breast** | **Gastrointestinal** | **Lung and Mediastinal** | **Hepato-biliary-pancreatic** | **Other cancers ^*^** | **P value** |
| --- | --- | --- | --- | --- | --- | --- | --- |
|  | **(N = 18)** | **(N = 7)** | **(N = 41)** | **(N = 39)** | **(N = 9)** | **(N = 8)** |  |
| **Surgery** | 5 (27.8) | 3 (42.9) | 28 (68.3) | 4 (10.3) | 2 (22.2) | 4 (50.0) | < 0.001 |
| **Radiotherapy** | 1 (5.6) | 0 (0) | 6 (14.6) | 9 (23.1) | 2 (22.2) | 1 (12.5) | 0.503 |
| **Chemotherapy** | 2 (11.1) | 5 (71.4) | 25 (61.0) | 33 (84.6) | 7 (77.8) | 5 (62.5) | < 0.001 |
| **Endocrine therapy** | 12 (66.7) | 5 (71.4) | 0 (0) | 0 (0) | 0 (0) | 1 (12.5) | < 0.001 |

Data are presented as number (percentage) and compared using Fisher’s exact test.

* Other cancers included gynecological cancer, thyroid cancer, oral cancer, head and neck cancer, sarcoma, and carcinoma of unknown primary.
