# Supplementary material for: Cardiology involvement and mortality in adult patients with advanced solid cancer complicated by atrial fibrillation
Source: PLoS One. 2025 Feb 25;20(2):e0319342. doi: 10.1371/journal.pone.0319342 (PMC11856317; doi:10.1371/journal.pone.0319342)
Supplement: S3 Table — (DOCX) [file pone.0319342.s006.docx]

**S3 Table. Causes of mortality in patients with AF and advanced solid cancer**

|  | **Without Cardiologist**  **(N = 51)** | **With Cardiologist**  **(N = 71)** |
| --- | --- | --- |
| **All-cause death** | 40 (78.4) | 44 (62.0) |
| **Cancer death** | 28 (54.9) | 37 (52.1) |
| **Cardiovascular death** | 5 (9.8) | 3 (4.2) |
| **Fatal myocardial infarction** | 0 | 0 |
| **Fatal stroke** | 0 | 0 |
| **Death due to heart failure** | 5 (9.8) | 3 (4.2) |
| **Death due to major bleeding** | 0 | 0 |
| **Others** | 7 (13.7) | 4 (5.6) |

Data are presented as number (percentage).

AF, atrial fibrillation.
