# Supplementary material for: Cardiology involvement and mortality in adult patients with advanced solid cancer complicated by atrial fibrillation
Source: PLoS One. 2025 Feb 25;20(2):e0319342. doi: 10.1371/journal.pone.0319342 (PMC11856317; doi:10.1371/journal.pone.0319342)
Supplement: Attachment — Submitted filename: Response to reviewers.docx [file pone.0319342.s007.docx]

**Response to Reviewer #1**

**Manuscript ID#:** PONE-D-24-53250R1

**Title:** Cardiology Involvement and Mortality in Adult Patients with Advanced Solid Cancer Complicated by Atrial Fibrillation

We thank the reviewer for reviewing our manuscript along with his/her invaluable suggestions upon our manuscript. In line with the comments, we have revised our manuscript. Our detailed responses will follow the reviewer’s comments. In addition, we revised the format and style of the manuscript to meet the PLOS ONE requirement. We also prepared a marked revision in which corrections in the text are marked in red to facilitate the review process.

**This is an interesting paper that retrospectively evaluated the prognostic impact of AF in solid cancers at a single center.**

**1. Heart failure as a comorbidity did not show any effect on prognosis. Is this related to the fact that atrial fibrillation did not show any effect on prognosis after adjustment for factors in patients with solid cancer?**

[Response]

Thank you for your important comment. We totally agree that heart failure and AF share some common backgrounds and they present a lot of interplays (e.g. heart failure might be a consequence of AF, *vice versa*). Therefore, as pointed out by the reviewer, the observation that heart failure as a comorbidity was not associated with survival might probably be related to the finding that AF was not associated with survival after multivariable adjustment in patients with solid cancer. In line with this comment, we added the following sentences in the revised Discussion section.

[Page 21, Bottom 2 lines – Page 22, Line 1]

Additionally, the presence of heart failure was not associated with mortality, either, further supporting that age, anemia, and oncologic features may have larger impact than cardiovascular comorbidities in such patients with advanced solid cancer.

**2. Could the involvement of the cardiology department have been a bias? For example, cancer patients with poor prognosis were not referred.**

[Response]

Thank you for this critical suggestion. We entirely agree with the reviewer that sicker patients with limited life expectancy might receive less referral to cardiologist, which is “confounding by indication” by definition. In the process of analysis, we did carefully adjust for covariates in order to minimize the effect of confounding. To this end, we constructed 3 multivariate models with age, comorbidities, and cancer types included as covariates, whose results on the association between cardiology involvement and reduced mortality were consistent. Hemoglobin and creatinine, as surrogates for “how sick the patient is”, were additionally adjusted in the revised manuscript according to Reviewer#2’s suggestion, which resulted in similar results with previous, making our observations more robust. However, we do admit that the unobserved confounders, including possible “confounding by indication”, might not be fully adjusted in these models. Therefore, in the Limitation section, we further added the following expression to be specific on “confounding by indication”.

[Page 23, Study limitations section, Lines 4-7]

Second, given the nature of observational studies, the observed association between cardiology involvement and mortality in patients with advanced cancer and AF may have been biased by unobserved confounders, including confounding by indication.

Finally, we again would like to thank the reviewer for the invaluable comments on our work, which have greatly helped us improve our manuscript. We sincerely hope that our further revised manuscript will be considered for publication in *PLOS ONE*.

**Response to Reviewer #2**

**Manuscript ID#:** PONE-D-24-53250R1

**Title:** Cardiology Involvement and Mortality in Adult Patients with Advanced Solid Cancer Complicated by Atrial Fibrillation

We thank the reviewer for reviewing our manuscript along with his/her invaluable suggestions upon our manuscript. In line with the comments, we have revised our manuscript. Our detailed responses will follow the reviewer’s comments. In addition, we revised the format and style of the manuscript to meet the PLOS ONE requirement. We also prepared a marked revision in which corrections in the text are marked in red to facilitate the review process.

**General comment**

**The authors showed that in patients with advanced solid cancer and atrial fibrillation, cardiology involvement is associated with reduced overall mortality. This is a very important finding, but what is the reason of this? Specifically, you showed that the cumulative incidence for neither cardiovascular nor non-cardiovascular death differed significantly between patients with and without cardiology involvement.**

[Response]

Thank you for this extremely important comment. As pointed out by the reviewer, in patients with advanced solid cancer and AF, although the overall mortality was lower in those with cardiology involvement, when we categorized the mortality into cardiovascular and non-cardiovascular deaths, neither differed significantly. However, we observed that both cardiovascular and non-cardiovascular deaths were numerically lower in those with cardiology involvement. The analysis for cardiovascular and non-cardiovascular deaths might have been underpowered due to the limited number of patients and events. These observations led to our speculation that the mechanism behind the reduced mortality might be attributed to the comprehensive evaluation of patients prompted by the multidisciplinary approach involving cardiologists. However, the reason is still unclear and requires future investigation.

In line with this comment, we revised the manuscript Discussion section as follows.

[Page 22, bottom 2 lines – Page 23, top 6 lines]

Both cardiovascular and non-cardiovascular mortality rates were numerically lower, but without statistical significance, in those with cardiology involvement compared to those without cardiology involvement. The limited number of patients and events, leading to the limited power of analysis, may have contributed to the lack of significant difference. The mechanism behind the reduced mortality, though remains unclear and warrants further investigation, could possibility be attributed to the comprehensive evaluation of patients prompted by the multidisciplinary approach involving cardiologists.

**Specific comment**

**1) The stage of the cancer would be related to the prognosis. Also, In the case of worse the cancer prognosis, it is possible that oncologists are less likely to consult with cardiologists, leading to the less chance to be involved by cardiologists.**

**Please provide the information of the stage of cancer between patients with and without cardiology involvement at the baseline in this study.**

[Response]

Thank you for this critical suggestion. We totally agree with the reviewer that the information on cancer stage should be included in this report, and added such information in the revised Tables 2 and 4 as follows. The comparisons showed that cancer stage was balanced between those with and without AF, and was also balanced between patients with and without cardiology involvement in the AF subgroup. Given that cancer staging system was different among different cancer types, we did not adjust for cancer stage in subsequent Cox analyses.

We also agree with the reviewer that sicker patients with limited life expectancy might receive less referral to cardiologist, which is “confounding by indication” by definition. Although we carefully adjusted for covariates in order to minimize the effect of confounding in the analyses, we do admit that the unobserved confounders, including possible “confounding by indication”, might not be fully adjusted in these models. Therefore, in the Limitation section, we further added the following expression to be specific on “confounding by indication”.

In line with these comments, we revised the manuscript as follows.

[Page 11, Table 1: the following rows were added]

|  | **All patients** | **Without AF** | **With AF** | **P-value** |
| --- | --- | --- | --- | --- |
|  | **(N = 1349)** | **(N = 1227)** | **(N = 122)** |  |
| **Staging of neoplasm** |  |  |  | 0.787 ^†^ |
| **Stage II ~ III** | 42 (3.1) | 38 (3.1) | 4 (3.3) |  |
| **Stage IV** | 1307 (96.9) | 1189 (96.9) | 118 (96.7) |  |

[Page 16, Table 3: the following rows were added]

|  | **Total patients with AF**  **(N = 122)** | **No**  **Cardiologist**  **(N = 51)** | **With**  **Cardiologist**  **(N = 71)** | **P-value** |
| --- | --- | --- | --- | --- |
| **Staging of neoplasm** |  |  |  | 0.639 ^†^ |
| **Stage II ~ III** | 4 (3.3) | 1 (2.0) | 3 (4.2) |  |
| **Stage IV** | 1307 (96.7) | 50 (98.0) | 68 (95.8) |  |

[Page 23, Study limitations section, Lines 4-7]

Second, given the nature of observational studies, the observed association between cardiology involvement and mortality in patients with advanced cancer and AF may have been biased by unobserved confounders, including confounding by indication.

**2) Comorbidities such as severe anemia and renal failure may be related to the prognosis of cancer patients. Please provide data of hemoglobin and creatine at the baseline. We also suggest additionally adjust for anemia and renal failure in the Cox proportional hazard model.**

[Response]

Thank you for this important suggestion. We agree that baseline hemoglobin and creatinine are essential variables to be displayed, as well as to be adjusted for in the multivariable models. Thus, we additionally included these two variables in the revised Tables 1 to 4, and adjusted for them in the multivariable Cox models. The additional adjustment for hemoglobin and creatinine in the Cox model for all-cause mortality in the entire cohort did not change the observation that AF was not associated with overall survival after multivariable adjustment (Table 2). The additional adjustment for hemoglobin and creatinine in the Cox model for all-cause mortality in the patients with AF did not change the observation that cardiology involvement was independently associated with lower mortality in such population, either (Models 1 to 3 in Table 4).

In line with this comment, we revised the manuscript as follows.

[Page 7, Statistical analysis, Para. 2, Lines 4-6]

The Cox proportional hazard model was adjusted for age, sex, hemoglobin, creatinine, hypertension, diabetes mellitus, stroke, cancer type, and cancer treatment.

[Page 7 bottom line – Page 8 top line]

Model 1 was adjusted for age, sex, AF classification, hemoglobin, creatinine, and comorbidities.

[Page 10, Table 1: the following rows were added]

|  | **All patients** | **Without AF** | **With AF** | **P-value** |
| --- | --- | --- | --- | --- |
|  | **(N = 1349)** | **(N = 1227)** | **(N = 122)** |  |
| **Hemoglobin (g/dL)** | 12.2 (10.6–13.6) | 12.2 (10.6–13.5) | 12.0 (10.1–13.9) | 0.913 |
| **Creatinine (mg/dL)** | 0.70 (0.57–0.87) | 0.69 (0.56–0.86) | 0.81 (0.68–1.00) | <0.001 |

[Page 12, Lines 8-10]

A positive correlation was observed between the presence of AF and mortality in crude analysis, which became neutral after adjusting for clinical characteristics (adjusted HR 1.08, 95%CI 0.84–1.39, p = 0.552) (Table 2).

[Page 15, Table 3: the following rows were added]

|  | **Total patients with AF**  **(N = 122)** | **No**  **Cardiologist**  **(N = 51)** | **With**  **Cardiologist**  **(N = 71)** | **P-value** |
| --- | --- | --- | --- | --- |
| **Hemoglobin (g/dL)** | 12.0 (10.1–13.9) | 12.3 (10.4–14.2) | 11.9 (10.2–13.6) | 0.159 |
| **Creatinine (mg/dL)** | 0.81 (0.68–1.00) | 0.79 (0.69–1.00) | 0.82 (0.68–0.99) | 0.791 |

[Page 2, Abstract, Results]

AF was associated with higher all-cause mortality, which became neutral after adjustment for age, sex, comorbidities, cancer types and cancer treatments (crude hazard ratio [HR] 1.39, 95% confidence interval [CI] 1.11–1.75, p = 0.004; adjusted HR 1.08, 95%CI 0.84–1.39, p = 0.552). In those with AF, cardiology involvement was independently associated with lower all-cause mortality (age, sex, comorbidities, cancer types and cancer treatments-adjusted HR 0.50 [95%CI 0.28–0.88], p = 0.017), though the cumulative incidence of neither cardiovascular nor non-cardiovascular death differed significantly between patients who received cardiology care and those who did not.

[Other revisions]

Tables 2 and 4, as well as the means and ranges of VIFs for multicollinearity listed in the manuscript text were also revised in accordance to the additional adjustment for hemoglobin and creatinine. Please refer to the Tables and text in the “marked revision” file for detailed corrections.

**3) Authors showed that anticoagulants were frequently administered to patients under cardiology care. What is the incidence of cerebrovascular disease (cerebral infarction and/or cerebral hemorrhage) between the two groups? Similarly, is there any difference of anemia between the two groups? It is expected that the presence of anemia will make it difficult to introduce anticoagulant therapy.**

[Response]

Thank you for this important suggestion. In both groups, no patients developed incident cerebral infraction or hemorrhage. One patient with cardiology involvement taking DOAC had traumatic subarachnoid hemorrhage.

Baseline comorbid anemia did not differ between groups as suggested by the comparable hemoglobin level in the revised Table 3, indicating that the increased prescriptions of anticoagulants in patients with cardiology involvement was independent from hemoglobin levels. Hemoglobin levels were then additionally adjusted for in the multivariable models, as stated in the response to the previous comment.

In line with these comments, we revised the manuscript as follows.

[Page 15, Table 3: the following rows were added]

|  | **Total patients with AF**  **(N = 122)** | **No**  **Cardiologist**  **(N = 51)** | **With**  **Cardiologist**  **(N = 71)** | **P-value** |
| --- | --- | --- | --- | --- |
| **Hemoglobin (g/dL)** | 12.0 (10.1–13.9) | 12.3 (10.4–14.2) | 11.9 (10.2–13.6) | 0.159 |
| **Creatinine (mg/dL)** | 0.81 (0.68–1.00) | 0.79 (0.69–1.00) | 0.82 (0.68–0.99) | 0.791 |

[Page 20, Para. 1, Lines 6-7]

In addition, no patient in either of the two groups had incident cerebral infarction or hemorrhage.

[Page 22, Lines 2-4 from bottom]

However, among the patients with advanced cancer, no patient had incident cerebral infarction or hemorrhage, and the cause of death was predominantly cancer, with only a small number of cardiovascular deaths.

**4) Is there a difference in prognosis between groups treated for AF and those not treated for AF among the patient with cardiologist involvement?**

[Response]

Thank you for this critical question. We additionally analyzed the group of patients with advanced solid cancer and AF who were cared by a cardiologist, and found that 66 (93%) out of 71 patients received either anticoagulant or antiarrhythmic (or both) as treatment for AF. The overall survival was similar between those who were and were not treated for AF among this subgroup of patients, as shown in the following Kaplan-Meier curves (log-rank p = 0.332). Given that the number and proportion of patients who did not receive any AF treatment in this subgroup was extremely small, we consider that the comparison between those with and without AF treatment as an exploratory analysis, and added this finding in the revised manuscript text as follows, without adding figures.

[Page 14, Para. 1, bottom 4 lines]

Additionally, among the 71 patients with AF and cardiology involvement, 66 received any AF treatment (either anticoagulant or antiarrhythmic or both), who presented comparable all-cause mortality with those who did not receive any AF treatment (log-rank p = 0.332).

**5) The figure numbers seem to differ from those in the manuscript (Figures 2-5).**

[Response]

We feel sorry for making such fundamental errors. The figure numbers are now corrected in the revised manuscript.

Finally, we again would like to thank the reviewer for the invaluable comments on our work, which have greatly helped us improve our manuscript. We sincerely hope that our further revised manuscript will be considered for publication in *PLOS ONE*.
